# Perceptions of supervision and feedback in PaedCompenda, the competency-based, post-graduate curriculum in pediatrics (www.paedcompenda.de)

**DOI:** 10.3205/zma001710

**Published:** 2024-11-15

**Authors:** Irene Somm, Marco Hajart, Folkert Fehr, Christoph Weiß-Becker

**Affiliations:** 1 Netzwerk Handlungsforschung und Praxisberatung, Cologne, Germany; 2 Gemeinschaftspraxis für Kinder- und Jugendmedizin, Sinsheim an der Elsenz, Germany; 3 Gemeinschaftspraxis für Kinder- und Jugendmedizin, Husum, Germany

**Keywords:** pediatrics, primary care, specialist training, learning, supervision, feedback

## Abstract

**Aim:**

Both teachers and learners had clear reservations in the beginning about the usefulness and benefits of supervision and feedback, which were to be implemented as a teaching method in the competency-based, post-graduate curriculum in general ambulatory pediatrics, known as PaedCompenda (www.paedcompenda.de). This paper investigates the different perceptions of the physicians undergoing specialist training (*Ärzte in Weiterbildung*) and elucidates these differences.

**Method:**

The following data were collected as part of the research on the three-year-long implementation (2019-2023):

1) Four focus group discussions (N=28) with physician trainees who had no experience in primary care pediatrics or with the post-graduate curriculum;

2) Problem-oriented interviews, one at the beginning and again at the end, with physician trainees (N=28) undergoing specialist training at 19 participating medical practices belonging to two post-graduate education networks;

3) Videos of patient consultations with the physician trainees (N=23);

4) Videos of feedback conferences regarding the videotaped patient consultations (N=7).

This data was evaluated using reconstructive grounded theory.

**Results:**

A distinctly more positive perception of the benefits of supervision and feedback as a teaching and learning method was seen in the physicians who received specialist training at the practices following the PaedCompenda curriculum. In regard to method, it is crucial that the educational setting can be experienced as a learning opportunity. Playing a central role in this is constructive and conducive feedback (a. dysfunctional routines, b. underlying lack of confidence, and c. overlooked problems).

**Conclusions:**

This paper shows the opportunities of an institutionalized form of supervision and feedback as part of a competency-based, post-graduate curriculum while also making it clear that implementation is challenging. Furthermore, the medical specialists who serve as trainers need to be specifically trained to know which approaches promote learning effectively.

## Zusammenfassung

**Zielsetzung:**

Gegenüber der Lehr-Lern-Methode von Beobachtung und Feedback, die im Rahmen des kompetenzbasiertes Weiterbildungscurriculum der ambulanten Allgemeinpädiatrie www.paedcompenda.de implementiert werden sollte, bestanden bei Lehrenden wie Lernenden anfänglich deutliche Vorbehalte hinsichtlich der Kosten-Nutzen-Relation. Im Beitrag werden differente Nutzenwahrnehmungen von Ärztinnen und Ärzten in Weiterbildung (ÄiW) untersucht und deren Unterschiede erklärt.

**Methodik:**

Folgende Daten wurden in der Begleitforschung zur dreijährigen Implementierung (2019-2023) erhoben:

1) Vier Fokusgruppen-Diskussionen (N=28) mit ÄiW ohne Erfahrung in der grundversorgenden Pädiatrie und mit dem Weiterbildungscurriculum,

2) Problemzentrierte Anfangs- und Schlussinterviews mit ÄiW (N=28) während der Praxis-Weiterbildung in 19 Projektpraxen zweier Weiterbildungsverbünde,

3) Videografierte Vorstellungsanlässe von ÄiW (N=23).

4) Videografierte Rückmeldegespräche zu aufgezeichneten Vorstellungsanlässen (N=7),

Die Daten wurden mit dem Ansatz der rekonstruktiven Grounded Theory ausgewertet.

**Ergebnisse:**

Bei ÄiW, die in den Praxen mit PaedCompenda weitergebildet wurden, kommt es zu einer deutlich positiveren Nutzenwahrnehmung der Lehr-Lern-Methode von Beobachtung und Feedback. In methodischer Hinsicht ist dabei entscheidend, dass das Setting als Lernanlass erlebt werden kann. Hier spielen förderliche Rückmeldeinhalte (a. dysfunktionale Routinen, b. unterschwellige Unsicherheiten und c. übersehene Auffälligkeiten) eine zentrale Rolle.

**Schlussfolgerungen:**

Der Beitrag zeigt zum einen die Möglichkeiten einer institutionalisierten Form von Beobachtung und Feedback im Rahmen eines kompetenzbasierten Weiterbildungscurriculums auf. Zum anderen macht er jedoch auch deutlich, dass die konkrete Umsetzung dieses Vorhabens herausfordernd ist und hierfür auch WBB gezielt dahingehend weitergebildet werden müssen, welche Vorgehensweisen tatsächlich lernförderlich sind.

## Introduction

The professionalization of post-licensure medical training in primary care pediatrics is being advanced using a digital, competency-based curriculum called PaedCompenda (PC) [https://www.paedcompenda.de/] [1]. However, despite broad approval, the implementation of this structured curriculum faces many obstacles which cannot be reduced solely to the notorious lack of time in the medical practices, but are also due to the lack of acceptance for the new teaching strategies accompanying the implementation of PC. At the start of a three-year-long research project (2019-2022) on the implementation of PC at 19 pediatric practices belonging to two model regions (Schleswig-Holstein and Mittelfranken), the feedback given by medical specialists to physician trainees directly after observing their interactions with patients was noticeably viewed with criticism, as a core teaching strategy, in terms of the time spent and the expected benefit.

This skepticism about the teaching method of *observation-based feedback* in the training of medical specialists is a known and much discussed issue. Many reasons have been identified in studies to explain this hesitation, primarily the unfavorable conditions of workplace-based learning in hospitals, a low quality of feedback, and the much too formal design of the observation and feedback procedures, particularly in the context of test-relevant assessments (e.g., Mini-CEX and DOPS) [2], [3], [4], [5], [6], [7], [8], [9], [10]. As a result, these implementation difficulties have generated numerous interventional studies. Weallans et al. [11] evaluate findings in an up-do-date review and identify empirically proven, but not yet sufficiently evidence-based, methodological components and principles that promise effective observation-based feedback in post-graduate learning environments.

The growing understanding that the effectiveness of feedback ultimately “depends on many variables, which overlap in individual cases and can inhibit or promote each other in their effects” [12] refers to the limits of interventional studies that investigate single variables derived from theory and obligate the test subjects, for the duration of the study, to follow an approach that is questionable in terms of sustainability because it is heavily regimented and independent from the situation.

Given this background, the medical practices participating in the implementation project were intentionally not required to follow a uniform approach. Although the teaching strategy was piloted in introductory seminars, when it came to implementation, each medical practice was called upon to seek a viable way that promised sustainability. Instead of prescribing a concrete approach from the outset, the initial focus was meant to be on the question central to this paper concerning the *trainees’ subjective perceptions of the qualitative differences in concretely experienced supervision and feedback*. Based on this, in a reversal, so to speak, of the method described above, content-specific and methodological components were *reconstructed* which, in their interactions, are able to explain the differences in the trainees’ perceptions of the benefits and usefulness. Following such an explanatory interpretive analysis [13], [14], [15], [16], it is possible, considering the findings of previous studies, to draw conclusions regarding the further development of this teaching strategy which does justice to the complex reality of individual instances of supervision and feedback.

## Method

The dataset, on which the results presented here are based, encompasses problem-oriented entrance and exit interviews [17], [18] with 28 physician trainees at 19 medical practices and four focus group discussions with 28 physician trainees at four different pediatric hospitals who had no experience with PC. In addition, six feedback conferences conducted by four trainers at the medical practices and 23 patient interactions with the physician trainees were videotaped. Feedback conferences lasting 60 to 90 minutes were held by the physician trainee, medical teacher, and researcher (see attachment 1 ). Verbatim transcripts were made of all audio and video recordings. The relevant questions guiding the interviews are contained in the attachment, although they also point toward other questions pursued by the research project (see attachment 2 ). The selection of the first 10 medical practices and their physician trainees was done pragmatically: Enquiries were made at practices in the German regions of Mittelfranken and Schleswig-Holstein that cooperated with hospitals via networks. The other data was collected afterwards, step by step, and followed the principles known in grounded theory as minimum and maximum contrast (among other things, the varying levels of experience amongst the physician trainees and the trainers). Even if this kind of systematic comparative analysis does not yield any statistically representative information, it does, however, enable *explanatory and interpretive* statements about an investigated subject [13], [14].

Data analysis was done using the computer program f4analyse in pursuance with reconstructive grounded theory [19]. This method is more consistent than traditional grounded theory in accounting for knowledge that is not reflexively available, as expressed in narratives and the description of experiences. In this way, it is possible to reconstruct the implicit learning [20] that has great significance for decision-making skills and responsibility [15], [16]. The coding procedure used is capable of differentiating between socially desirable or singular statements and those statements with a high degree of personal relevance pointing to the heart of the matter concerning the benefits focused on here. Statements made by the physician trainees were coded consistently in the context of meaning of the entire interview. Furthermore, central text passages underwent detailed sequential analysis (see examples in attachment 3 ), in which coding was done not only in terms of content, but also in regard to form: *how and in which context something is said*.

This analytical method does not allow for a purely descriptive presentation of results, meaning that the separation of description and interpretation, as established in quantitative social research, cannot be adequately converted into a qualitative form of social research. This is because the findings are made on the basis of interviews, the meanings of which cannot be simply “read” from the literal meanings of the words, but rather require a methodologically controlled process of interpretation [19].

## Results

**Figure 1:**
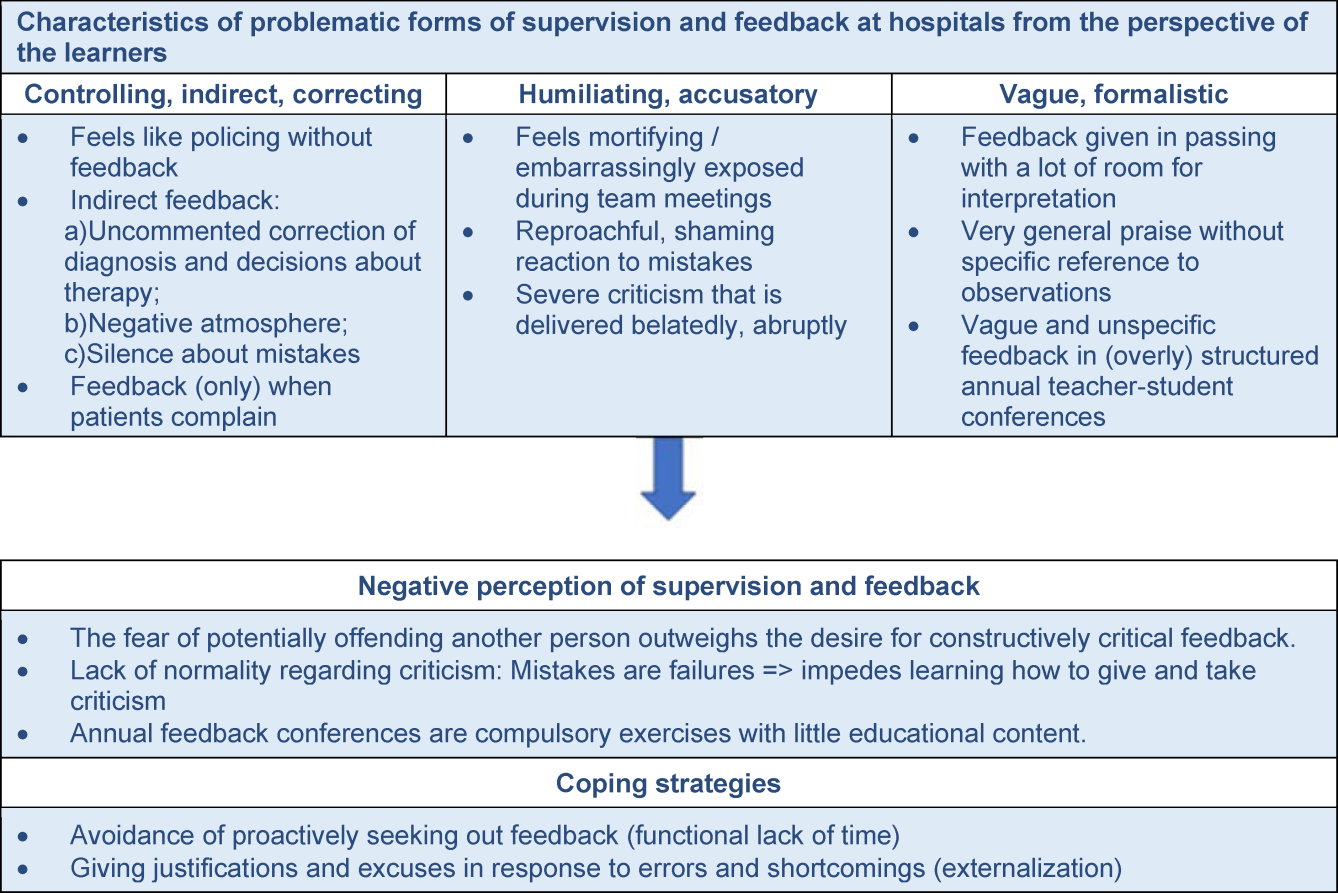
Negative perceptions of supervision and feedback (Data: Physician trainees at four pediatric hospitals without post-graduate experience at a medical practice (n=28))

in the physician trainees at the participating practices about being supervised and then receiving feedback. Although feedback, particularly constructively critical feedback, had been missed in previous post-graduate training, their hesitation was still impossible to overlook when it involved actually implementing the teaching method. To explain these reservations, their experience with receiving feedback in the hospital setting was examined more closely. Overall, it was noticeable that descriptions of problematic or absent feedback predominated; only four of 56 physician trainees with hospital experience reported receiving feedback that explicitly enhanced their learning during their post-graduate training. As a result, it was possible to reconstruct key characteristics and aspects of problematic experiences with supervision and feedback that lead toward a *negative perception*. At the same time there were coping strategies by the physician trainees identified that cemented their lack of acceptance for situations involving supervision and feedback. A summary of these findings is presented in figure 1 (Fig. 1).

Over the course of implementing PaedCompenda at the medical practices, the level of acceptance for the teaching method has *improved* in all of the surveyed physician trainees. However, a distinction must be drawn between an unreservedly positive perception and a *less positive perception* of the benefits. In the following, findings are presented that justify these distinctions.

### Less positive perceptions at the participating medical practices

**Figure 2:**
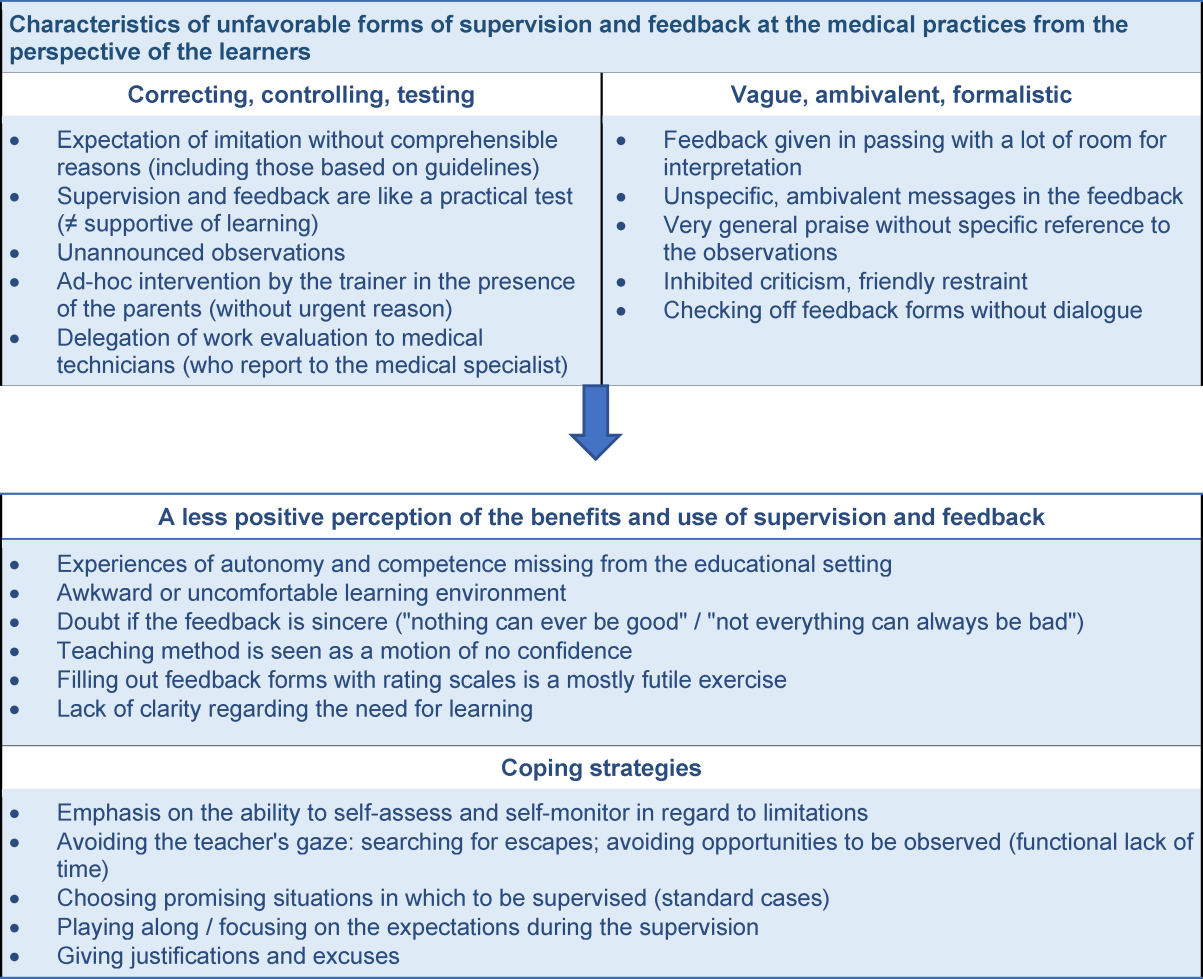
Less positive perceptions (Data: Physician trainees at the participating medical practices using supervision and feedback according to PaedCompenda (n=28, each with two interviews))

Figure 2 (Fig. 2) lists characteristics and aspects of feedback experiences leading to a *reduction in the positive perception* in the physician trainees at the medical practices, along with typical coping strategies, in the same manner as figure 1 (Fig. 1). It is impossible to address all of these aspects within the scope of this paper. Those which are covered in detail in the following represent a *deliberate selection* from the larger picture and involve aspects which have not previously received much attention in the literature.

#### Expectations of being imitated without justifiable reason

One type of feedback which was experienced negatively by the physician trainees at the medical practices involves an *incomprehensible or almost incomprehensible expectation of imitation*. Specifically, when the recommended corrections in the trainer's feedback do not align with the guidelines or evidence-based recommendations and no further justification is given for this deviation. Such eminence-based, rather than evidence-based, judgements [21] promote “playing along” behavior in situations of supervision, as is made clear in the following statement:

*In fact, if I know that the one supervisor is present, then I do the work more like he wants it done. And when the other supervisor is there, then I do it a little more like she wants it done. If I am alone, I choose somewhere in-between the two that makes sense to me (MF 6).*

This behavior is heightened if trainers position themselves as irrefutable role models in the way that they give feedback and become very much caught up in a paradigm of right or wrong [22]; when *“this is how I do it”* turns into *“this is how it is done”*. As a result, specifically advanced and self-confident physician trainees feel themselves restricted in their autonomy, an aspect that in the field of education generally is a central factor of sustained motivation to learn [23]. In response, these trainees emphasize their skill at assessing themselves and their ability to self-monitor, hence relativizing the benefits of being observed and receiving feedback.

#### Unspecific and ambivalent feedback

Similarly frustrating for physician trainees is feedback that lacks specificity and/or feedback in the form of taciturn statements that open up a *wide scope for interpretation* which, depending on personal disposition, tend to be interpreted either in a self-serving or a self-critical way, as is shown in the following example:

*Interviewer: Did she (the trainer) then give some feedback at the end?*

*Interviewee: Not explicitly. (Both laugh.) I mean, she did not say: “You did that well”. But then I did – what one is only so happy to do in these cases – I said something like: “Yeah, well, I was suddenly really nervous when you were looking over my shoulder at what I was doing”. And in her reply, it was possible to make out that she thought everything was okay. (SH 13)*

The feedback from the trainer in this example is specifically not explicit and the consequence is that, although the physician trainee *was able* to “make out” some message in the trainer’s reaction, one could also say that the trainee was required to do precisely this.

In the videotaped feedback conferences, it was also seen that the trainers routinely followed up critical comments with a kind of relativization, in which they emphasized that what was happening here was really just *complaining about nothing truly important*. As a result, the criticism took on a distinctly ambivalent character – something that would rather be hidden or only handled with kid gloves. This also corresponds to the fear often expressed by the trainers in the feedback workshops that their critical comments will compromise or discourage the trainees.

In such an atmosphere of inhibited criticism, a natural culture of observation and feedback can hardly be established: Physician trainees primarily remember the situations where they were observed and received feedback as an *uncomfortable learning environment* with limited benefits. As a consequence, these physician trainees attempt to avoid the trainer's attention and, in this context, the purported lack of time is only too useful for steering clear of such situations.

### Positive perceptions at the participating medical practices

**Figure 3:**
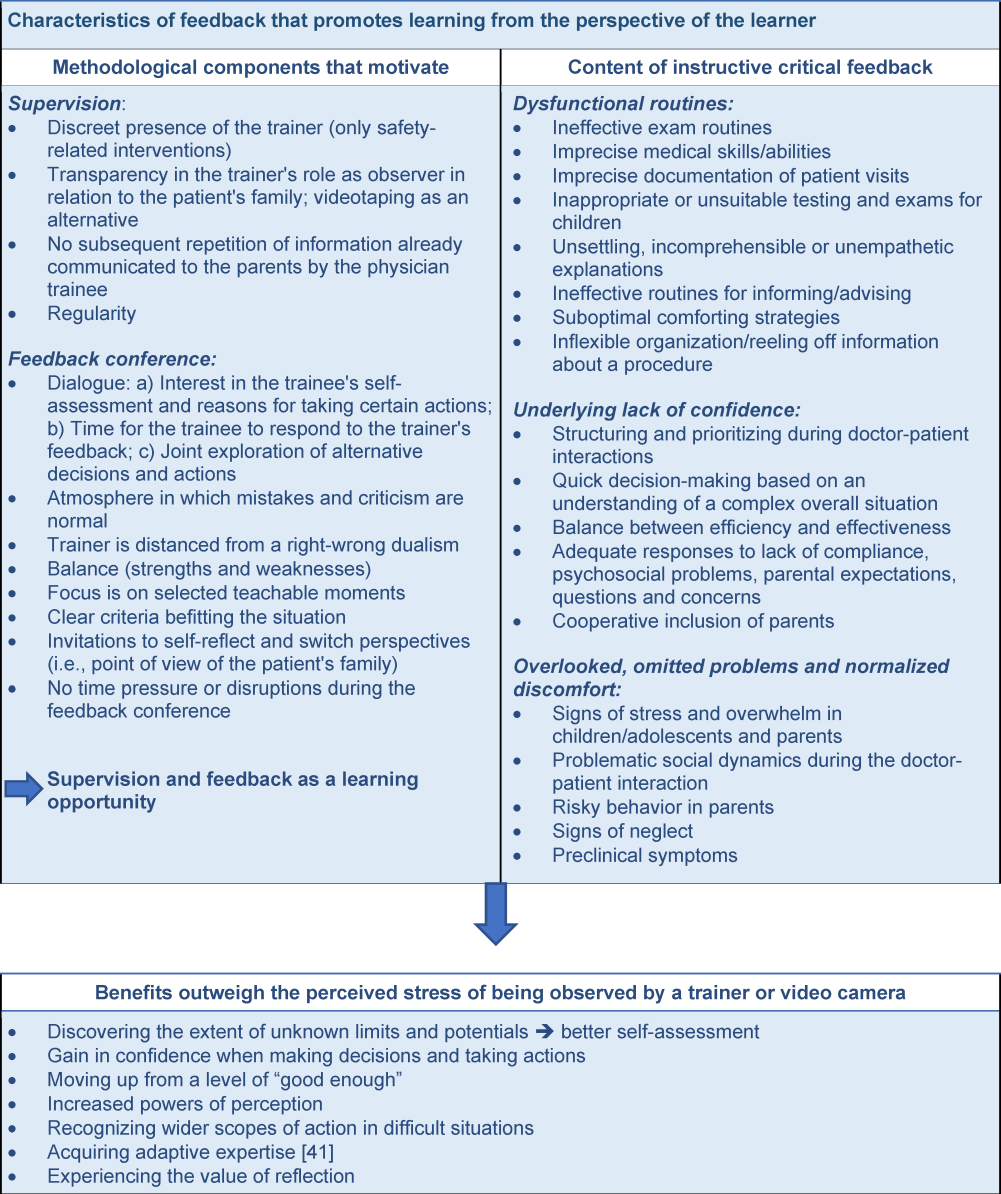
Positive perceptions (Data: Physician trainees at the participating medical practices with experience in direct supervision and feedback (n=28, each with two interviews))

The methodological and content-based features of observation-based feedback favoring an unreservedly positive perception of its benefits are presented in figure 3 (Fig. 3). Regarding the *methodological components*, it is possible to summarize so: If feedback from the trainer is informed mainly by an atmosphere of learning and not a performance evaluation, then the desire *“to do it perfectly for the colleague”* can fade into the background:

*I am just not always good and that’s the reason I am still learning. And even once I have learned everything, there are probably still areas where I make mistakes or things that I can do better. And, yes, that is actually the hardest point, to unlearn that, to think about how you make mistakes and embarrass yourself. (SH 6)*

This statement reveals how hard it is for physicians to separate themselves from the internalized expectation of infallibility. If trainers are successful in enabling the physician trainees to experience *critical* feedback primarily as a *learning opportunity* and not as a source of shame or poor evaluation, then perceptions will become distinctly more positive. Such an experience mainly occurs when the supervision is focused specifically on separate teachable moments and if there is a two-way dialogue exploring the desired and undesired effects of the medical work that was observed. Very often this is not about whether the treatment administered during a medical consultation was right or wrong, but rather the ways in which the quality of care could be increased. Or conversely, which approaches/measures taken by the physician trainee were particularly effective. Some trainers even used this as an opportunity to scrutinize their own practices and thereby showed themselves to be open to learning. The latter makes it easier for novices to step outside of the right/wrong dualism [22] mentioned above and let go of the* “fear of doing something wrong”*.

In terms of *content*, these kinds of teachable moments are especially distinguished by a sense that they enable a *conscious formation of previously and reflexively inaccessible limits and potentials of one’s own actions* (for an example, see the fine analysis in attachment 3 ). The following goes into brief detail regarding the three central characteristics listed in figure 3 (Fig. 3).

#### Feedback on dysfunctional routines

Feedback addressing the suboptimal routines acquired and unquestioningly reproduced by physician trainees in the first few years of their professional practice is described as very helpful:

*There are so many things, beginning with set phrases that one has gotten used to and doesn’t really notice, but also medical details or finer points or imprecisions that have crept in. Or when explaining to patients: How do I explain the use of a therapy or medication, something that I, myself, don’t quite realize I am doing with imprecision or maybe in a way that isn’t easy to understand. (SH 4)*

Based on these insights, the physician trainee quoted above judges the supervision of patient-doctor interactions by his trainer as *“one of the best parts”* of his training at the medical practice. The reason for his positive assessment lies predominantly in the identification of his routine’s unintended effects. Looking back, the routines acquired in the hospital setting are sometimes even described as a kind of *“muddling through”*. At any rate, whenever physician trainees receive this type of feedback, they are able to see that in a variety of areas they persistently remain at a “good-enough level” because this kind of mentorship was absent from the learning process.

#### Feedback on underlying lack of confidence

Much of the uncertainty that physician trainees feel at the beginning of their specialist training at a medical practice can be dispensed with by gathering increased experience and having the opportunity to consult with specialist practitioners when needed. That said, there are other insecurities which are unacknowledged or unconscious or considered not important enough that they need to be articulated – and as a result remain hidden. Physician trainees view it as extremely beneficial when these underlying issues are focused on in the feedback from observing their work and discussed with the aim of finding solutions. It is not seldom that, precisely on these occasions, competencies they were unaware of get mirrored back to them and this contributes to building confidence. In terms of topics, this often involves a lack of confidence in adequately structuring the medical consultation given specific case details, in quicker and more prudent processes of clinical reasoning, and in the competencies associated with holding conversations with patients (for more details: see figure 3 (Fig. 3)).

#### Feedback on overlooked or omitted problems

The random nature of patient consultations in primary outpatient care poses an often unexpected challenge for physician trainees. It demands of them a wide-ranging perspective on case histories and diagnoses and, likewise, a heightened alertness for subclinical physical, emotional and cognitive symptoms in children. Furthermore, they must also take notice of the conduct and behavior exhibited by parents. Feedback on supervised situations showing physician trainees where they have overlooked a problem, or even ignored it, is experienced as a clear gain in the required skill of perceiving patients accurately:

*For example, we had the situation where a mother picked the child up by her hands so that it could sort of sit there, but then she couldn’t really sit down herself, and it all looked like something that was routinely done. Then he drew my attention to it by saying, “Look at this situation…”." I had seen it but hadn’t registered it that way. That was then very helpful and you can learn pretty simply what things you need to pay a bit of attention to. (SH 12)*

It is significant that “seeing” and “registering” are described in this quote as being two different things. A common insecurity is made visible here: How does what is seen impact a physician's actions during the complex event of a patient-parent interaction? If appropriate feedback does not come from the trainer, what the physician trainee has seen often remains without consequence and is thus occluded. These and similar normalizations of discomfort and irritation were seen frequently on the videotapes of early detection exams (see attachment 1 ). Situations which were certainly perceived as problematic, even unsettling, were responded to with previously learned routines and not seen as unusual or unexpected situations that asked for a flexible response tailored to fit the individual case. When such situations are recalled during the feedback conference and reflected upon together in search of alternatives, then the judgement about the benefits of being supervised and receiving feedback are unreservedly positive:

*Being supervised makes total sense, 100 percent. As stupid as it is, but again, afterwards, those two videos: It happens a lot, when I am alone in a consultation, screening a patient, I am reminded of things....I find that super important. (MF 1)*

In this statement, the physician trainee refers to detailed feedback on videotapes of patient consultations. In general, it is seen that by watching the videos together, it is possible to very successfully increase one's powers of perception and then realize that there is a need to learn.

## Discussion

In the following, focus is placed on the question of how a positive perception of supervision and feedback and, hence, an increase in acceptance of this teaching and learning method could be encouraged in physician trainees. These considerations corroborate and underscore previous findings and recommendations in the literature.

First, the results emphasize a now widely stated recommendation, according to which observation-based feedback in medical education and post-licensure training should be designed as an assessment for learning rather than an assessment of learning [10]. With this, the trainees *learning goal orientation* [24] should be strengthened, while their *performance goal orientation* [24] fades into the background [2], [25], [26]. That precisely this has relevance in highly regimented, performance-based educational systems is shown by analyses of the learning culture in medicine [10], [27], whereby the critical discussion predominantly centers on the feedback settings in the context of test-relevant evaluations (summative feedback). For example, after many years of experience with Mini-CEX, the Royal College judged that feedback content “is often lacking, ineffective, excessively positive and commonly avoids negative aspects” [28]. In the German-speaking countries, also, studies contain many indications of a limited perception of the usefulness or benefits, for instance, due to the excessive formalization and standardization of feedback procedures [3], [5], [29]. In response to this, a variety of educational institutions have modified their evaluation forms and replaced a reductionist, scale-based mode of scoring with an open-ended, criteria-guided form of evaluation [29], [30]. Our three-year-long research project came to this same conclusion: The original feedback forms [31] were revised based on the SIWF form [29]. One aim was to formulate general evaluation criteria which encompass the range of expectations – medical, organizational and communicative-specific to general ambulatory pediatrics [12] (cf. attachment 4 ). The sources for this evaluative “anchor” [29] are guidelines, textbooks on primary pediatric care [32], and analyses of recorded patient consultations [27]. Given a context in which trainers impose expectations of imitation that physician trainees can find hard to comprehend, as described above, these criteria ideally provide a corrective measure against individual instances of eminence-based feedback [21]. This function can be significantly supported through the regular sharing of information on standards of good outpatient care in quality circle meetings for trainers, as they have been implemented in the field of pediatrics.

Also consistent with other studies is our finding that, based on observed patient consultations, a *friendly correction of one’s self-estimation/self-image or a productive disturbance of the conviction that one has sufficient ability to self-assess* contributes to the perception of the effectiveness of feedback [33]. It has been found numerous times that the development of adequate self-assessment requires professional assessment by an outside person [2], [34], [35]. Our results support the conclusion reached by Jünger et al. [34], in which instructive feedback must mainly aim to *identify unconscious competencies and deficits*.

The extent to which the three *content-based* characteristics we identify in meaningful feedback can be generalized needs further empirically clarification, as the question regarding the instructive content in feedback has so far remained mostly unexamined (cf. [36]). However, bearing in mind the literature on learning theory, there are indications that the* feedback on dysfunctional routines* and the* feedback on underlying lack of confidence* described here increases the perceived instructive content of the feedback beyond the scope of general ambulatory pediatrics. For example, the concept of “deliberate practice” [37], [38] makes it clear that frequent repetition of activities to gain a better routine is not sufficient in itself to develop a qualitatively high degree of expertise. Rather this demands the *realization of unfavorable automatisms* that block improvement. Taken within the context here, this means that “deliberate practice” by physician trainees begins when previously learned routines are questioned in terms of their merit and when insecurities that arise while interacting with patients are not dispensed with by normalizing them, but rather understood to be a call for continued learning. It makes sense that this requires a conducive learning environment and professional mentorship that supports a desire to reflect [39], [40]. Although the time factor plays a crucial role, it is not sufficient by itself. This requires trainers who do not act like irrefutable role models [22], but rather use the training of young physicians to ponder their own practices and, in this respect, to experience supervision and feedback as a source of inspiration for themselves.

The third focal area, described above (*omitted and overlooked problems*, see figure 3 (Fig. 3)) and perceived as highly beneficial, affects medical activities connected with primary prevention specifically. Nonetheless, a more generalizable competency dimension is also present that can be strengthened through observation-based feedback: In addition to correctly applying “routine expertise” in familiar situations, this involves the ability to develop “adaptive expertise” to deal with novel or unexpected situations [40], [41]. Although learners are at first tasked with acquiring confidence by developing and training their routine knowledge and abilities, they should still learn early on that a strictly schematic application of medical rules is not always appropriate or conducive [42]. Because they will regularly – as shown above – find themselves confronted with situations that can only be dealt with effectively if they are not perceived as familiar standard situations, but rather met with adaptive measures (cf. the anamnesis example: [43]). In our experience, fostering this kind of cognitive flexibility [44] is particularly successful when feedback is given on videotaped patient consultations because the slowed-down viewing of the events allows the complexity of the situation to become visible and the question of how to apply expertise in a way that is adapted to the case and the situation is asked almost automatically. Previous studies also confirm that video-recordings as part of the feedback have a potential to differentiate the feedback’s content and increase its perceived usefulness [45], [46], [47], [48]. As of now, it has been rather uncommon to investigate in more detail what is required of the trainers. According to what we observe, productive reflections on a videotape also entail genuine interest on the part of the trainer concerning “deliberate practice” [37] and an associated no-blame culture that encourages and promotes learning [49]]. This aspect should certainly be the focus of further study. Summarized in table 1 (Tab. 1) are several recommended formats derived from the results for training the trainers and mentors who, according to our experience, can support a learning-focused feedback culture.

### Limitations

This paper draws on the experience-based perceptions of the physician trainees. As a result, characteristics and processes of supervision and feedback come into view that explain the subjectively perceived differences in quality. However, based on the selected research design, the extent to which these differences in the perception of benefits or usefulness are, in fact, also reflected in modified action still remains open. This would require, for example, a systematic and longitudinal observation of real patient interactions for many physician trainees in combination with the corresponding feedback conferences with their trainers in order to reconstruct potential connections.

The sampling was limited to medical practices that had not been randomly selected for the pilot project; rather, it was more the case that the trainers voluntarily chose to participate. A certain self-selection can be assumed in terms of motivation and possibly also a self-assumed competency on the part of the trainers regarding observation-based feedback. Nevertheless, the results contained examples of more positive perceptions of the benefits and usefulness and also less positive ones. The requirement for theoretical (as opposed to statistical) representativeness, in which an investigated phenomenon should be explored as comprehensively as possible through a sample with regard to its various theoretical aspects, can therefore nevertheless be considered as fulfilled here. 

Likewise, it can be asserted that the reservations mentioned in the introduction regarding supervision and feedback certainly cannot be placed solely on the shoulders of the physician trainees. As indicated throughout the paper, trainers also face the question of how they are going to handle a situation and which obstacles may exist on their part when attempting to implement ways to give supportive feedback. This needs to be looked at more closely in further studies.

And finally, considering the always situated and context-dependent experiences of the trainees, the question arises to what extent the findings from the pediatric context can be generalized to other medical areas or even to feedback processes as a whole.

## Notes

### Adherence to ethical standards

The authors state that there is no conflict of interest. The ethical standards were adhered to. The studies were carried out in compliance with national law and the Declaration of Helsinki from 1975 (in the current revised version). GDPR-compliant consent to the collection of all data is available.

### Funding

The accompanying research project on learning processes in the network for post-graduate education in pediatrics received funding from the Schleswig-Holstein Ministry for Social Affairs, Health, Youth, Family, and Seniors (*Ministerium für Soziales, Gesundheit, Jugend, Familie und Senioren des Landes Schleswig-Holstein*) and the PaedNetz-Mittelfranken e.V.

### Authors’ ORCIDs

- Irene Somm: [0000-0001-9969-461X]
- Marco Hajart: [0000-0003-3537-5868]
- Folkert Fehr: [0000-0001-8495-4167]

## Competing interests

The authors declare that they have no competing interests. 

## Supplementary Material

Dataset

Interview guidelines

Detailed sequential analysis 1 of supervision/feedback using SH 6 as an example

Supervision and feedback in pediatric primary care

## Figures and Tables

**Table 1:**
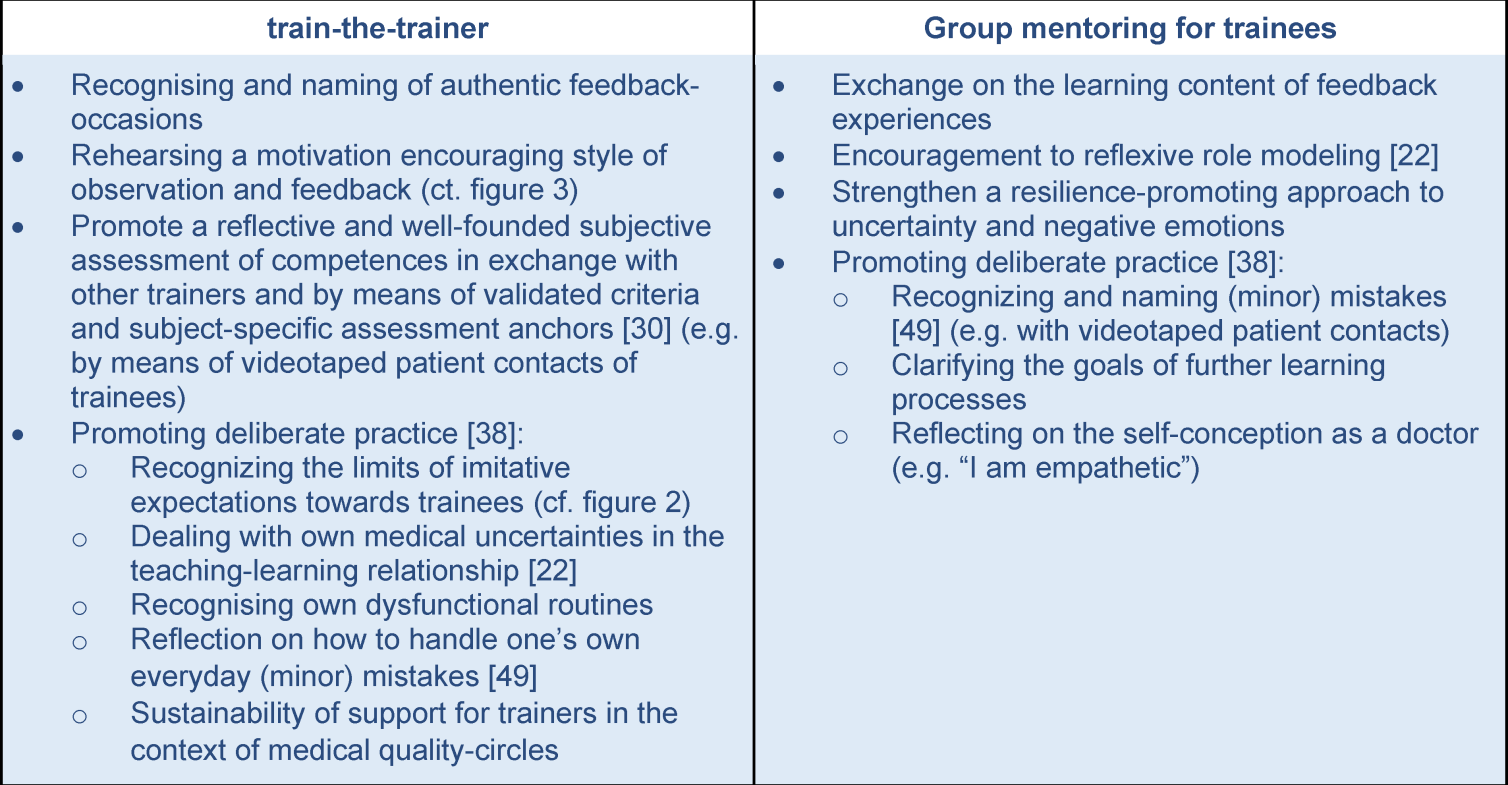
Recommendation of practical application
