# Supplementary material for: Perceptions of supervision and feedback in PaedCompenda, the competency-based, post-graduate curriculum in pediatrics (www.paedcompenda.de)
Source: GMS J Med Educ. 2024 Nov 15;41(5):Doc55. doi: 10.3205/zma001710 (PMC11656182; doi:10.3205/zma001710)
Supplement: Dataset [file JME-41-55-s-001.pdf]

Attachment 1: Dataset

Problem-oriented entrance and exit interviews with physician trainees at medical practices (2019-2022)

Medical practices	Physician trainee	Year of training	Duration/extent of training in a medical practice
Mittelfranken I, group practice (GP)	MF 1, f	5th	12 months, 100%
Mittelfranken II, individual practice (IP)	MF 2, f	5th	12 months, 100%
Mittelfranken III, GP	MF 3, f	4th	12 months, 50%, 30% hospital
Mittelfranken VI, GP	MF 4, f<br>MF 6, m	3rd<br>3rd	14 months, 100%<br>12 months, 100%
Mittelfranken V, GP	MF 5, f<br>MF 7, f		12 months<br>12 months
Schleswig-Holstein I, GP	SH 2, f<br>SH 3, f<br>SH 9, f<br>SH 16, m	5th<br>2nd<br>4th<br>3rd	6 months, 50%, 30% hospital<br>24 months, 50%<br>12 months, 100%<br>12 months, 100%
Schleswig-Holstein II, GP	SH 1, f	5th	12 months 50%, 30% hospital
Schleswig-Holstein III, GP	SH 4, m<br>SH 14, f	4th<br>?	12 months, 100%<br>10 months, 50%
Schleswig-Holstein IV, GP	SH 5<br>SH 12		12 months, 50%, 50% hospital<br>15 months, 50%, 30% hospital
Schleswig-Holstein V, GP	SH 6, m<br>SH 15, f<br>SH 17, m<br>SH 18, f	2nd<br>3rd<br>3rd<br>4th	24 months, 50%, 50% hospital<br>10 months, 50%<br>26 months, 100%<br>18 months
Schleswig-Holstein VII, GP	SH 7	2nd	24 months, 100%
Schleswig-Holstein VIII, IP	SH 8	4th	10 months, 50%
Schleswig-Holstein IX, GP	SH 10, f	3rd	18 months, 50%
Schleswig-Holstein X, GP	SH 21, f	3rd	24 months, 50%
Schleswig-Holstein XI, GP	SH 11, f	4th	13 months, 50%
Schleswig-Holstein XII, GP	SH 13, f	4th	12 months, 50%
Schleswig-Holstein XIII, GP	SH 19, m	2nd	12 months, 100%
Schleswig-Holstein XIV, GP	SH 20, f	4th	12 months, 50%

Attachment 1 to Somm I, Hajart M, Fehr F, Weiß-Becker C. *Perceptions of supervision and feedback in PaedCompenda, the competency-based, post-graduate curriculum in pediatrics (www.paedcompenda.de)*. GMS J Med Educ. 2024;41(5):Doc55. DOI: 10.3205/zma001710

Partially standardized focus group discussions (2019-2020)

Hospitals	Physician Trainees	Year of training
Mittelfranken I	7 participants, 6 f, 1 m	1st through 5th
Mittelfranken II	3 participants, 2 f, 1 m	3rd through 5th
Schleswig-Holstein I	8 participants, 8 f, 2 m	1st through 5th
Schleswig-Holstein II	9 participants, 8 f, 3 m	1st through 5th

Videotaped feedback conferences (2019-2022)

Practice/Training experience	Physican trainee	Occasion
SH I, 12 years	f, 5th	Early detection exam "U4"
SH 3, 11 years	m, 2nd<br><br>f, 4th	Early detection exam "U5"<br>Acute care I, II<br>Early detection exam "U4"
SH 20, 3 years	f, 3rd	Early detection exam "U8"
SH 19, 11 years	m, 3rd<br>m, 3rd	Provision of vaccination information<br>Acute care I

Videotaped patient consultations with physician trainees (2020-2022)

Physician trainee	Year of training	Occasion
MF 1	5th	Early detection exams "U9", "U3"
MF 2	5th	Early detection exam "U8"
MF 3	4th	Early detection exams "U3", "U6"
MF 4<br>MF 6	4th<br>5th	Early detection exams "U9", "U8", "U5"<br>Early detection exam "U8"
SH 1	5th	Early detection exam "U8"
SH 6	2nd	Early detection exam "U5" Acute care I, II
SH 8	4th	Early detection exam "U7"
SH 9	4th	F Early detection exam "U4"
SH 12	3rd	Early detection exam "U8"
SH 13	4th	Early detection exam "U4"
SH 16	4th	Early detection exam "U8"
SH 19	2nd	Vaccination advice
SH 20	3rd	Early detection exams "U5", "U8"
SH 21	3rd	Early detection exams "U8", "U9"

Attachment 1 to Somm I, Hajart M, Fehr F, Weiß-Becker C. *Perceptions of supervision and feedback in PaedCompenda, the competency-based, post-graduate curriculum in pediatrics (www.paedcompenda.de)*. GMS J Med Educ. 2024;41(5):Doc55. DOI: 10.3205/zma001710
