# Supplementary material for: Perceptions of supervision and feedback in PaedCompenda, the competency-based, post-graduate curriculum in pediatrics (www.paedcompenda.de)
Source: GMS J Med Educ. 2024 Nov 15;41(5):Doc55. doi: 10.3205/zma001710 (PMC11656182; doi:10.3205/zma001710)
Supplement: Interview guidelines [file JME-41-55-s-002.pdf]

Attachment 2: Interview guidelines

A: Problem-oriented interview I with the physician trainees (after approximately 3 weeks of specialist training at the medical practice)

	Guided questions/impetus for discussion	***Subtopics/aspects***	***Follow-up questions** (only as needed, immanent questions first!)*
**Start**	How did you come to decide to spend a year at a pediatric medical practice? (reword to match length of time)<br>How did you choose the medical practice?<br>You have been at the practice now since **.<br>How is the work going?<br>What do you find valuable?<br>What do you find not so valuable?	• Motives/reasons for specialist training at a medical practice (explicit but also implicit ones!)<br>• Priorities regarding choice<br>• Initial assessment of the work (exploration of relevant evaluation criteria)	• How did you find out about the post-graduate training network?<br>• What was said about it?<br>• In which areas did you work at the hospital?
**Initial phase**	How did the first weeks go, exactly?<br>In which areas do you already work independently? How did that come about?<br>What was new at first for you at the pediatric medical practice? (Ask for examples.)<br>What main things have you learned in the past weeks?<br>Were there situations during the past weeks where you thought: Oh no, what am I doing?	• Specific practice-based training in the first weeks<br>• Content/basis of the independence<br>• Initial/current sources of irritation or discomfort<br>• Expected/unexpected need for learning<br>• Self-assessment	• How long have you shadowed? At what kind of patient appointments? Conference afterwards?<br>• Was there anything unexpected for you?<br>• Where do you still feel uncertain when giving acute care?<br>• What is similar to the emergency room? What is different?<br>• Were you able to directly apply what you learned in emergency medicine to the work at the medical practice?

Attachment 2 to Somm I, Hajart M, Fehr F, Weiß-Becker C. *Perceptions of supervision and feedback in PaedCompenda, the competency-based, post-graduate curriculum in pediatrics (www.paedcompenda.de)*. GMS J Med Educ. 2024;41(5):Doc55. DOI: 10.3205/zma001710

**Quality of specialist training**	What do the interactions with your trainer look like?<br>Over the past weeks what were the questions that you often asked your trainer?<br>What have been the benefits of shadowing your trainer at the medical practice? (Examples)<br>How do you experience the interactions with your trainer?<br>What is particularly helpful? What isn't?<br>When you watch the trainer, are there things you would do differently?	• Experiences with superiors/trainer<br>• Assumption of responsibility for tasks?<br>• Confidence in one's own competencies; trust in the trainer's competencies<br>• Commitment of the trainer<br>• Relationship<br>• Reflexive role-modeling<br>• Benefits of shadowing and asking questions	• Which tasks/patient cases would you be happy to take independent responsibility for in addition?<br>• Are there (subject) areas in which you still feel (more or less) uncertain? Are there other tasks that were assigned to you before you felt confident?<br>• I am going to assume for a moment that you have also made a mistake at some point. What was that like for you? How did your superiors handle it?<br>• Do you do some things differently than your boss?
**PaedCompenda**	What is your experience with PC?<br>How do you use the lists of learning objectives?<br>Was that helpful? In what way?<br>You said that you are still uncertain about *. Did the lists of learning objectives with the linked book help you?<br>Do you receive feedback? From whom? How and in which situations?<br>What do the supervision and feedback look like?<br>What was it like to be observed?<br>What was commented on? (Examples)<br>Did it benefit you?	• Perception/assessment of the usefulness and benefits of the teaching methods (also in comparison to shadowing and asking questions)<br>• Feedback regarding competencies/deficiencies<br>• Relevance of the structuring<br>• General conditions for implementation	• What basic conditions are necessary to implement PC?<br>• How do you recognize if or to what extent you are performing your work well/correctly? (feedback/supervision culture)<br>• What things indicate to you if and to what extent you are becoming better or more competent than your trainer over the course of your post-graduate education?
**Outlook**	What would you most like to learn in the coming months? (potential discussion about rarely mentioned topics/areas)<br>How could/should the trainer at the practice improve further? What is needed for that?	• Awareness of the need for learning<br>• Relevant actions for desired changes	

B: Problem-oriented interview II with the physician trainees (2-3 weeks before completion of specialist training at the medical practice or shortly thereafter)

	Guided questions/impetus for discussion	***Subtopics/aspects***	***Follow-up questions** (only as needed, immanent questions first!)*
**Start: Example cases**<br>(For examples, see focus group guidelines)	To begin with, I would like to show you two example cases in general ambulatory pediatrics.<br>What thoughts spontaneously go through your head when you read them?<br>What would you do if you were a practicing pediatrician?<br>Which questions need to be asked to assess the situation appropriately?<br>a) First case<br>b) Second case<br>Have you seen similar cases?	• Setting the focus: (medical) diagnosis vs. (also) parental worries/concerns<br>• Systemic view<br>• (Child-centered) procedural approach?<br>• Problematization vs. normalization<br>• Cooperative approach<br>• Understanding of medicine<br>• Understanding of responsibility<br>• Relevant actions depending on positions taken regarding the presented case	• What advice would you give the parents?<br>• How do you think the situation will develop further?<br>• Would it make sense to involve other professionals (preschool, midwife, speech therapist, etc.)?<br>• Is this even a relevant problem which you, as a pediatrician, are "responsible" for dealing with?
**Review**	When you look back: What did you especially like at the medical practice? What less so?<br>What would you have liked to have learned even better?<br>Specifically bring up individual aspects from the first interview and ask about further developments!	• Teachable moments<br>• Criteria for meaningful medical care<br>• Self-assessment	• Was there anything you lacked?<br>• What was communicated about that?<br>• In which areas did you work at the hospital?

**Benefits of specialist training at a medical practice**	Was it a good decision to go to a medical practice? Why?<br>Can you take what you've learned and apply it in the hospital setting? (Ask for examples)<br>How do you view the idea of introducing a medical practice rotation for all physician trainees?	• Educational content/benefit/added value	• Will you work differently at the hospital in the future? (If so, in what way?)
**Quality of post-graduate education/PaedCompenda**	What type of support from the trainer and other colleagues was especially helpful and instructive? (Ask for examples)<br>In the first interview, you mentioned that you speak with the trainer about *. Was this possible?<br>In retrospect, what was lacking for you?<br>What does PaedCompenda deliver? (Discuss individual tools, ask for examples, and go into detail.)	• Assessment of and experience with different teaching and learning methods/tools<br>• Circle back to assessments in the first interview (changes? improvements? changes for the worse?)<br>• General conditions of / time invested in post-graduate education	• Where do you see a need for improvement regarding PaedCompenda? (Go into detail.)
**Outlook**	Where will you go next? Hospital? A medical practice? Open your own practice? Take employment? Senior Physician at a hospital?	• Future prospects<br>• Obstacles<br>• Work-family balance/Work-life balance	• How, where, and in what position would you like to work after completing your medical residency?

C: Guidelines for the focus group discussion with physician trainees at hospitals

	Guided questions/impetus for discussion	***Subtopics/aspects***	***Follow-up questions** (only as needed, immanent questions first!)*
Topic block 1**: Experiences with pediatricians in private practice / with general pediatrics in the outpatient setting*<br>Topic block 2:** Ideas about / images of ambulant primary care*	What experiences have you already gathered in the hospital regarding collaboration with pediatric practitioners?<br>➔ Ask for specific examples!<br>What do you think the daily routine at an ambulatory pediatric practice looks like?<br>What are the pros and cons of a specialist training phase at a medical practice?	• Expectations about practicing physicians from the perspective of someone working at a hospital<br>• Assessment criteria<br>• Notions about the competency profile in ambulatory pediatric practice<br>• Reasons for and against a specialist training phase at a medical practice	• What expectations do you have regarding collaboration with pediatricians in private practice?<br>• Are there aspects of the collaboration that ought to be improved?<br>• Are there also things about the collaboration that anger you?<br>• In your view, what abilities are necessary to be a "good" primary care pediatrician?<br>• In your opinion, what makes for a good primary care pediatrician?
***Topic block 3**: Post-graduate education*	How is post-graduate training currently organized at your hospital?<br>What works well? What doesn't work so well?<br>How do you recognize if or to what extent you are performing your work well/correctly?<br>In what form do you receive feedback?<br>I am going to assume for a moment that you have also made a mistake at some point. How was that for you? How did your superiors handle it?	• Degree of structure/desire for structure<br>• Quality of mentorship/desire for mentorship<br>• Experiences with superiors/post-graduate instructors<br>• Feedback regarding competencies/deficiencies<br>• Confidence in one's own competencies; trust in the instructor's competencies<br>• Dealing with uncertainty<br>• Error culture, no-blame culture	• How and by whom are you mentored?<br>• Are there (subject) areas in which you still feel (more or less) uncertain? Are there other tasks that were assigned to you before you felt confident?<br>• Are there (subject) areas that you think can be better learned at a medical practice?<br>• What do wish were different in respect to your post-licensure training?<br>• What things indicate to you if and to what extent you are becoming better or more competent than your instructor over the course of your post-graduate education?<br>• Would you be happy taking on the responsibility for other/more tasks?<br>• What do you do when you feel uncertain?

***Topic block 4**: Example cases*	I would like to show you two example cases from primary care pediatrics.<br>What thoughts go through your head?<br>What is your take on the situation?<br>How would you approach this?<br>Which questions need to be asked?<br>What advice do you give?<br>a) First case (see below)<br>b) Second case (also below)	• Setting the focus: (medical) diagnosis vs. (also) parental worries/concerns<br>• Systemic view<br>• (Child-centered) procedural approach?<br>• Problematization vs. normalization<br>• Cooperative approach<br>• Understanding of medicine<br>• Understanding of responsibility	• What advice would you give the parents?<br>• How do you think the situation will develop further?<br>• Would it make sense to involve other professionals (preschool, midwife, speech therapist, etc.)?<br>• Is this even a relevant problem which you, as a pediatrician, are "responsible" for dealing with?<br>• Similar cases? (Example 1)

Example case 1

Baby Paul, 5 weeks old, is brought for the first time to the pediatrician's office. The parents report that Paul cries for hours every day, especially in the evening, and can hardly be calmed down. He wants to be nursed constantly, but then feeds only briefly. If he falls asleep while being held and the mother carefully places him in bed, he wakes up immediately and begins to cry again. This means that the mother carries Paul around all day in her arms and can't get anything done. When he comes home in the evenings, Paul's father carries him around too. When nothing else works, he will take Paul out in the car for a drive. Then Paul stops screaming and falls asleep peacefully. He mainly sleeps well at night starting around 11 p.m. and only needs to be nursed once.

Several evenings ago they took Paul to the emergency room at the local pediatric hospital because Paul had screamed particularly loudly and they had become worried that the child really might have an internal problem. The pediatrician examined Paul thoroughly, arranged for an ultrasound of his abdomen and a blood test. Everything came back normal. The pediatrician reassured them that Paul is completely healthy, just that he is probably one of those babies who cry incessantly.

Their midwife has shown them how best to hold and rock Paul and how they should massage his stomach. She recommended liquid drops for wind. The maternal grandmother remembers that Paul's mother was also a very restless baby and cried a lot. She recommended putting Paul in his crib and letting him cry. The parents are emotionally unable to do this. The parents are exhausted; they never could have imagined that a baby could be such a strain. They desperately need advice about how they can calm Paul down. Paul

was a planned child. He was born following an uncomplicated pregnancy with a birth weight of 3600 g, length 51 cm, cranial circumference 36 cm. The family is healthy. Paul is breastfed only and gaining weight well; weighing 4300 g today. The clinical findings are normal.

Example case 2

Five-year-old Tarik appears for the routine "U9" early detection exam. The parents are from Turkey; Tarik was born in Germany. The family speaks Turkish. The family lives in a neighborhood where the inhabitants are predominantly Turkish and Arab. Tarik has attended preschool for 3 years. During the visit, the mother asks for a prescription and referral for speech therapy because the child stood out at the medical check for school enrollment as having insufficient proficiency in German. According to the information given by the mother, Tarik speaks Turkish well. When speaking with the child, many grammatical errors are noticeable, but the boy can understand and make himself understood well and has a large vocabulary. A hearing test a couple of weeks ago indicated normal hearing.

(Case examples supplied by the German General Ambulatory Pediatrics Association (Deutsche Gesellschaft für ambulante Allgememeinpädiatrie (DGAAP))
