# Supplementary material for: Perceptions of supervision and feedback in PaedCompenda, the competency-based, post-graduate curriculum in pediatrics (www.paedcompenda.de)
Source: GMS J Med Educ. 2024 Nov 15;41(5):Doc55. doi: 10.3205/zma001710 (PMC11656182; doi:10.3205/zma001710)
Supplement: Detailed sequential analysis 1 of supervision/feedback using SH 6 as an example [file JME-41-55-s-003.pdf]

Attachment 3: Detailed sequential analysis 1 of supervision/feedback using SH 6 as an example

Interviewer: And how was that for you? You said that it is new.

B: That is new, yes.

Confirmation of the implied change

It is, in any case, still very unfamiliar

Necessary, unavoidable process of habituation and adaptation

, because he is also critical

Adaptation does not involve being supervised, but rather the content of the feedback

Unaccustomed criticism; absence of critical feedback in prior (three-year-long) post-licensure training in the hospital?

. That is, he very clearly identifies the things that he thinks are not good,

Articulated vs. unarticulated criticism

Uncertainty about the quality of the work performed at the hospital (what isn't thought to be good is not identified)?

but also what he thinks is good.

Same: articulated praise vs. unarticulated praise

And since being observed is unfamiliar.

Secondary aspect of habituation and adaptation to the new learning method

Naturally, you struggle with it a bit,

Unpleasant situation (sensation is felt in the body - directly perceptible) ==> therefore:

Obviousness of potentially avoidant behavior (escape reflex)

(see other text passages: "not having time" is used as an avoidance strategy)

Relativizing the negative

but then you come to agree

Admission, need to revise one's own self-assessment or conviction; willingness to view things differently,

Attachment 3 to Somm I, Hajart M, Fehr F, Weiß-Becker C. *Perceptions of supervision and feedback in PaedCompenda, the competency-based, post-graduate curriculum in pediatrics (www.paedcompenda.de)*. GMS J Med Educ. 2024;41(5):Doc55. DOI: 10.3205/zma001710

, that it is simply, yes, it is the best training, basically.

description using the superlative; doubtlessly positive evaluation of the learning effect (new conviction)

Training: practice, repeat, routine => in context: supervised training: gain in "critically supervised" routine

Also: routine practice in being observed? A new aspect to become used to

Or to receive such direct feedback,

Immediacy, clarity of the feedback

these are of course also things, when you think back on your time studying at university, that just happened so seldom simply because so little practical instruction takes place and very little feedback is given.

Reference to medical study: retrospective judgment about what was lacking

And that is really something, it is hard to always hang in there, in the daily routine of a busy medical practice.

Restrictions of daily routine work in a medical practice; regularity is compromised

But that is where you are certainly going to learn the most and take it with you.

Repeated use of the superlative in regard to the benefit of the learning method

Well, to be observed really closely by the mentor and then get feedback right away. #00:19:36-0#

Individual interview. SH4, lines 48 - 49

Summarizing notes

The challenge of establishing a routine at the medical practice where the training is received and the discomfort of being supervised do not detract in any way from the very positive evaluation. Both are obstacles that, from the point of view of the physician trainee, once overcome enabled the learning effect this trainee experienced.

Attachment 3 to Somm I, Hajart M, Fehr F, Weiß-Becker C. *Perceptions of supervision and feedback in PaedCompenda, the competency-based, post-graduate curriculum in pediatrics (www.paedcompenda.de)*. GMS J Med Educ. 2024;41(5):Doc55. DOI: 10.3205/zma001710
