# Supplementary material for: Perceptions of supervision and feedback in PaedCompenda, the competency-based, post-graduate curriculum in pediatrics (www.paedcompenda.de)
Source: GMS J Med Educ. 2024 Nov 15;41(5):Doc55. doi: 10.3205/zma001710 (PMC11656182; doi:10.3205/zma001710)
Supplement: Supervision and feedback in pediatric primary care [file JME-41-55-s-004.pdf]

Attachment 4: Supervision and feedback in pediatric primary care

Attachment 4 to Somm I, Hajart M, Fehr F, Weiß-Becker C. *Perceptions of supervision and feedback in PaedCompenda, the competency-based, post-graduate curriculum in pediatrics (www.paedcompenda.de)*. GMS J Med Educ. 2024;41(5):Doc55. DOI: 10.3205/zma001710

Supervision and Feedback

In the context of PaedCompenda, instructors regularly receive observation-based feedback. This feedback relates to specific tasks/behaviors in daily practice and is based on criteria. This ensures that the evaluation is transparent and understandable.

Tipp: During the observation, instructors should remain in the background and only intervene in exceptional cases. The more often such supervisions occur, the more natural they become. At first, they may feel unfamiliar to all involved.

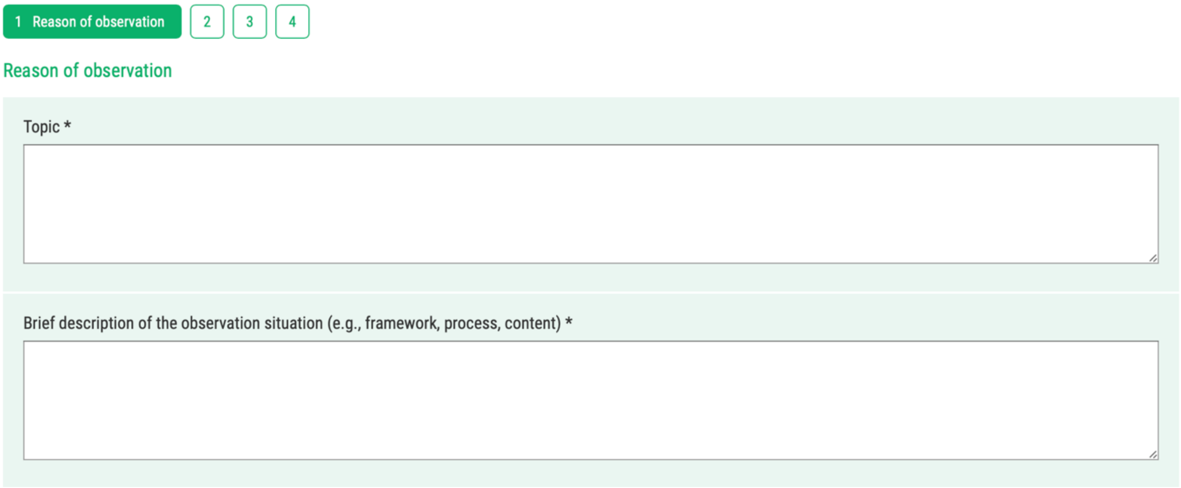
1 Reason of observation 2 3 4

Reason of observation

Topic *

Brief description of the observation situation (e.g., framework, process, content) *

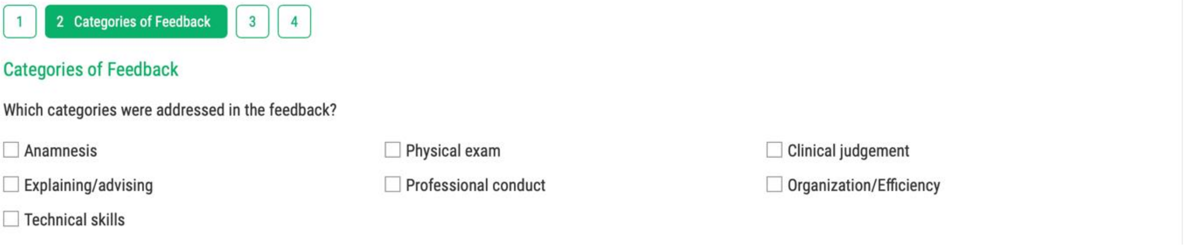
1 2 Categories of Feedback 3 4

Categories of Feedback

Which categories were addressed in the feedback?

- ☐ Anamnesis
- ☐ Physical exam
- ☐ Clinical judgement
- ☐ Explaining/advising
- ☐ Professional conduct
- ☐ Organization/Efficiency
- ☐ Technical skills

1 | 2 | 3 Contents of the feedback discussion | 4

Contents of the feedback discussion

What was positive (see evaluation criteria)? *

What can be improved? *

What do we aim to do? *

Other remarks (teaching needs etc.) *

Evaluation criteria

Evaluation criteria

Anamnesis

- Asks focused questions, tailored to the clinical picture or the issue, and pays attention to red flags.
- Asks open-ended and closed questions focused on the patient's reason for coming to the doctor
- Actively listens and detects risks by asking empathetic follow-up questions.
- Utilizes interpreters or data protection-compliant translation apps in a timely manner.

Physical exam

- Follows an efficient and logical sequence, adapts the exam to the child's condition and age and the reason for the visit;
- Explains the exam procedure to the patient's family;
- Includes the gathering of evidence if there is a suspicion of maltreatment/abuse.

Clinical judgement

- Accurately assesses the severity of the disease;
- Recognizes complications and emergencies and quickly refers the patient;
- Considers differential diagnoses when there is reason to do so;
- Creates a clarification and therapy plan while considering the concerns of the patient's family.
- Arranges for appropriate diagnostics, as needed;
- Communicates the findings and decisions within the team clearly and understandably
- Informs the family in an understandable manner and responds appropriately to concerns

Explaining/Advising

- Explains in an evidence-based, balanced and understandable manner; takes individual information needs into account
- Explicitly obtains consent for the further course of action; specifies the conditions for follow-up
- Makes use of (multilingual) informational materials
- Avoids moralizing and patronizing behavior;
- Explores any difficulties following through if there is non-adherence

Professional Conduct

- Shows empathy and respect
- Responds appropriately and constructively to dissent
- Correctly assess one's own skills; asks for help when needed
- Observes the duty of confidentiality

Organization/Efficiency

- Works in a structured and efficient manner; keeps adequate records;
- Shows flexibility when handling unforeseen situations;
- Deals with other topics in additional consultations
- Knows the legal framework

Technical skills

- Performs interventions correctly, skillfully, and precisely
- Pays attention to avoidable painful procedures
- Achieves a satisfactory result
- Works in a sterile manner, avoiding external and self-harm
- disposes of materials properly
